# Decrease in Blood Pressure and Regression of Cardiovascular Complications by Angiotensin II Vaccine in Mice

**DOI:** 10.1371/journal.pone.0060493

**Published:** 2013-03-27

**Authors:** Futoshi Nakagami, Hiroshi Koriyama, Hironori Nakagami, Mariana Kiomy Osako, Munehisa Shimamura, Mariko Kyutoku, Takashi Miyake, Tomohiro Katsuya, Hiromi Rakugi, Ryuichi Morishita

**Affiliations:** 1 Department of Clinical Gene Therapy, Graduate School of Medicine, Osaka University, Suita, Osaka, Japan; 2 Department of Geriatric Medicine, Graduate School of Medicine, Osaka University, Suita, Osaka, Japan; 3 Division of Vascular Medicine and Epigenetics, United Graduate School of Child Development, Osaka University, Suita, Osaka, Japan; University of Illinois at Chicago, United States Of America

## Abstract

Vaccines have been recently developed to treat various diseases such as cancer, rheumatoid arthritis and Alzheimer’s disease in addition to infectious diseases. However, before use in the clinical setting, vaccines targeting self-antigens must be demonstrated to be effective and safe, evoking an adequate humoral immune response from B cells while avoiding T cell activation in response to self. Although the vaccine targeting angiotensin II (Ang II) is efficient in rodents and humans, little is known regarding the immunological activation and safety of the vaccine. In this study, we evaluated the efficiency and safety of an Ang II peptide vaccine in mice. Immunization with Ang II conjugated to keyhole limpet hemocyanin (KLH) successfully induced the production of anti-Ang II antibody, which blocked Ang II signaling in human aortic smooth muscle cells. However, Ang II itself did not activate T cells, as assessed by the proliferation and lymphokine production of T cells in immunized mice, whereas KLH activated T cells. In an Ang II-infused model, the non-immunized mice showed high blood pressure (BP), whereas the immunized mice (Ang II-KLH) showed a significant decrease in systolic BP, accompanied by significant reductions in cardiac hypertrophy and fibrosis. Importantly, anti-Ang II antibody titer was not elevated even after the administration of large amounts of Ang II, indicating that Ang II itself boosted antibody production, most likely due to less activation of T cells. In addition, no accumulation of inflammatory cells was observed in immunized mice, because endogenous Ang II would not activate T cells after immunization with Ang II-KLH. Taken together, these data indicate that vaccines targeting Ang II might be effective to decrease high BP and prevent cardiovascular complications without severe side effects.

## Introduction

Although vaccines are common therapies to prevent infectious diseases, they have recently been expanded to treat diseases such as cancer, rheumatoid arthritis and Alzheimer’s disease by targeting self-antigens.[1]–[6] For example, the amyloid beta vaccine effectively reduced amyloid plaques and recovered memory functions in several animal models of Alzheimer’s disease.[3]–[5] Unfortunately, however, the clinical trial of this vaccine was halted when 6 % of the participants developed aseptic meningoencephalitis, despite amyloid plaque reduction in the patients.[4], [7] The postmortem examination of the brains of two patients who suffered from aseptic meningoencephalitis due to the vaccine revealed T lymphocyte infiltration into the brain.[8], [9] This finding may suggest that the adverse effects of the vaccine were due to a T-cell-mediated autoimmune response.[4], [8]–[10] This theory is also supported by the presence of a T-cell epitope in the amyloid beta used for immunization, which was considered responsible for eliciting autoimmunity. Consequently, the vaccine was modified to exclude T-cell epitopes, thereby avoiding T-cell activation without disrupting the B-cell epitopes responsible for antibody production.[11]

In contrast, vaccines for hypertension, targeting the renin-angiotensin system, have been reported since the 1950s_ENREF_12. [6], [12]–[23] A renin vaccine was reported to successfully reduce blood pressure (BP).[15]–[22] However, after Michel *et al.* reported that the vaccine induced autoimmune disease of the kidneys in two animal models,[21], [22] no further research on the renin vaccine was reported. An angiotensin I (Ang I) vaccine also reduced BP in rat and mouse models.[12], [23] Nevertheless, the vaccine did not reduce BP in the clinical trial.[13] The reason for the failure was considered to be the feedback pathway between angiotensin II (Ang II) and renin. In contrast, an Ang II vaccine was reported to be effective at producing anti-Ang II antibodies in both rodents[14] and humans.[6] However, little is known regarding the safety, especially in terms of its mechanism of action, because the report only showed the reversibility of the antibody titer and no immune complex deposition or inflammation. For a vaccine to be efficient, it is generally accepted that the antibodies it induces should decrease the concentration of their target through binding and clearance[1]. Unexpectedly, this mechanism does not explain the efficiency of the Ang II vaccine, because this vaccine reduces BP, despite increased Ang II levels in immunized rats compared to controls.[14] Thus, in this study, we further evaluated the efficacy and safety of the Ang II peptide vaccine.

## Materials and Methods

### Peptide syntheses

Keyhole limpet hemocyanin (KLH) is a standard carrier protein that is immunogenic and contains a strong T-cell epitope.[24] KLH is derived from the limpet, which is only distantly related to mammals. Therefore, KLH contains only non-self T-cell epitopes. KLH (Wako Pure Chemical Industries, Osaka, Japan) was conjugated to the N-terminus of Ang II using glutaraldehyde (Peptide Institute Inc., Osaka, Japan). BSA was conjugated to the N-termini of Ang II and Ang I using suberic acid bis (Peptide Institute Inc., Osaka, Japan). The protein concentrations of the peptides were determined using the Bradford assay.

### Immunization protocol

Nine-week-old male C57/BL6J mice and eight-week-old male SHRs (spontaneous hypertensive rats) were purchased from The Oriental Yeast Company (Osaka, Japan) and housed in a temperature- and light cycle-controlled facility. These experiments were approved by the Ethical Committee for Animal Experiments of the Osaka University Graduate School of Medicine. Various doses of the peptide solution were mixed with an equal volume of Freund’s adjuvant (Wako Pure Chemical Industries) prior to the injections and emulsified; 100 µl of the mixture was subcutaneously injected into mice. The antigen dose corresponded to the dose of Ang II in Ang II-KLH. The vaccination protocol consisted of three injections at two-week intervals. Priming was performed with complete Freund’s adjuvant, and the boosts were performed with incomplete Freund’s adjuvant. Serum was isolated from the tail vein and subjected to an ELISA. Some mice were euthanized on day 42, and their spleens were excised for ELISPOT and T-cell proliferation assays. The other mice were used as the Ang II infusion model.

For SHR experiments, vaccination protocol was almost similar. Twenty-four week-old male rats were utilized in this study. The injected antigen was 5 µg per rat with Freund’s adjuvant three times on day0, 14 and 21 after immunization.

### T cell proliferation assay

T-cell proliferation assays were performed, as previously reported.[25], [26] Mouse splenocytes (10^6^ cells/well) were re-stimulated in vitro with 10 µg/ml recombinant Ang II, angiotensinogen (Sigma-Aldrich, Tokyo, Japan), KLH, phytohemagglutinin (PHA) (Wako Pure Chemical Industries) or Ang II-KLH conjugate. The cells were incubated for 36 hours, and 1 µCi of [^3^H]-thymidine (Perkin Elmer, Japan) was added to each well for 12 hours. The cells were harvested, and the [^3^H]-thymidine uptake (counts per million, cpm) was determined using a MicroBeta 1450 TriLux scintillation counter (Wallac Oy). The stimulation index is expressed as the ratio of stimulated cells to non-stimulated cells.

### Detection of cytokine production using ELISPOT

To detect the production of the lymphokines, IFN-γ and IL-4, we used re-stimulated splenocytes isolated from the experimental mice, as previously reported.[25] Briefly, 96-well ELISPOT plates (Millipore, Tokyo, Japan) were coated with anti-mouse IFN-γ or IL-4 antibody, as recommended by the manufacturer (R&D Systems, Tokyo, Japan). After an overnight incubation, the wells were blocked with 1.5 % BSA and 5 % sucrose. The splenocytes from individual immunized mice were seeded in wells (10^6^ cells/well) and restimulated with 10 µg/ml recombinant Ang II, angiotensinogen, KLH or the AngII-KLH conjugate. After 48 hours of incubation (37 °C, 5 % CO_2_), the plates were washed five times with PBS-T. Then, the wells were incubated with biotinylated anti-mouse IFN-γ or IL-4 antibody overnight at 4 °C. After washing with PBS-T, a streptavidin-alkaline phosphatase complex was added, and the cells were incubated for 2 hours at room temperature. After washing with PBS-T, BCIP/NBT solution was added to the wells for color development, followed by incubation for 30 minutes at room temperature. The plates were washed with deionized water and air-dried overnight. The colored spots were evaluated using a dissecting microscope (Olympus, Tokyo, Japan).

### Enzyme-linked immunosorbent assay (ELISA)

The ELISA was performed as previously reported.[12] Ang II-BSA conjugate, Ang I-BSA conjugate or angiotensinogen was coated on ELISA plates (MaxiSorp Nunc, Thermo Fisher Scientific K.K., Japan) to measure the specific antibody titers for Ang II, Ang I and angiotensinogen. The peptides at 5 µg/ml were coated onto plates in carbonate buffer overnight at 4 °C. After blocking with 3 % skim milk in phosphate-buffered saline (PBS), the sera were diluted 1∶100 to 1∶10,000,000 in blocking buffer and incubated at 4 °C overnight. In the competitive ELISA, the sera were diluted 1∶1,000, pre-incubated with various doses of recombinant Ang II at room temperature for 30 minutes and applied to the coated plate. After washing with PBS-0.05 % Tween (PBS-T), the plate was incubated with horseradish peroxidase (HRP)-conjugated antibodies specific for mouse IgG (GE Healthcare, Tokyo, Japan) for 3 hours at room temperature. After washing, the color was developed using 3,3′,5,5′-tetramethylbenzidine (TMB) solution (Sigma-Aldrich), and the reaction was stopped using 0.5 N sulfuric acid. The absorbance was read using a microplate reader (Bio-Rad Inc., Japan). The endpoint titer is expressed as the serum dilution that exhibited half-maximal binding.

### Neutralizing Ang II-induced ERK phosphorylation

Human aortic smooth muscle cells (HASMCs) were purchased from Clonetics Corp. (Palo Alto, CA) and were maintained as previously described. The cells were cultured in smooth muscle basal medium (SmBM) (Lonza, Tokyo, Japan) and supplemented with 100 U/ml penicillin and 100 µg/ml streptomycin. All of the cells were cultured at 37 °C in 5 % CO_2_. Recombinant Ang II was pre-incubated with sera from the control (1 mg KLH/mouse) or immunized mice (1,000 ng Ang II-KLH/mouse) on day 42 for 30 min at 37 °C. The cells were treated with pre-incubated Ang II (10^−7^ M) (Sigma-Aldrich) for 10 min, and western blot analysis was performed as previously described.[27] The total protein was extracted from the cells using lysis buffer (50 mmol/L Tris-HCl, 2.5 mmol/L EGTA, 1 mmol/L EDTA, 10 mmol/L NaF, 0.1 % sodium deoxycholate, 1 % Triton X-100, 1 mmol/L PMSF, and 1 mmol/L Na_3_VO_4_), size-fractionated using SDS-PAGE, and transferred to an Immobilon-P membrane (0.45 µm pore size)(Millipore, Bedford, MA). The membrane was incubated overnight with antibody against pERK or ERK (Cell Signaling Technology K.K., Tokyo, Japan) at 4 °C. After washing, the membrane was incubated with an HRP-conjugated antibody (GE Healthcare, Tokyo, Japan) and washed. The blot was developed using ECL Plus western blotting detection reagents (GE Healthcare). The signal levels were visualized using an LAS-1000 Plus gel documentation system (Fujifilm, Tokyo, Japan).

### Neutralizing Ang II induced c-fos promoter activity

The c-*fos* promoter assay was performed as previously described.[28] Briefly, HASMCs were transfected with a c-*fos* luciferase reporter gene (p2FTL) using Lipofectamine 2000 Transfection Reagent (Invitrogen, Carlsbad, CA) The c-*fos* luciferase reporter gene consisted of 2 copies of the c-*fos* 5′-regulated enhancer element (−357 to −276), the herpes simplex virus thymidine kinase gene promoter (−200 to 70), and the luciferase gene. Recombinant Ang II was pre-incubated with 1 % sera from control (1 mg KLH per mouse) or immunized mice (1000 ng AngII-KLH per mouse) for 30 min at 37°C. At 24 hours after transfection, cells were stimulated with the pre-incubated Ang II (10^–7^M) for 24 hours. The cells were subsequently washed with PBS, and lysed with cell lysis buffer (Promega, Japan). A total of 20 µl of the cell extract was mixed with 100 µl of the luciferase assay reagent, and the luminescence of the resulting mixture was measured using a luminometer (Berthold, Japan).

### Ang II infusion model

Continuous Ang II infusion was performed as previously reported.[29] On day 42, osmotic mini-pumps (Alzet®) containing Ang II (1 µg/kg/min) were implanted into mice. On day 49, blood pressure was measured using the tail-cuff method (Softron Co, Tokyo, Japan). The mice were eutanized on day 56, their hearts were excised for histological examination and mRNA was collected for reverse transcription-polymerase chain reaction.

### Histological Examination

The hearts were fixed in 4 % formalin overnight, embedded in paraffin, and sectioned into 5-µm-thick slices at the middle-level of the capillary muscles. The heart sections were stained with Masson trichrome to assess fibrosis. The fibrotic area was quantified as previously reported[30] using ImageJ software (version 1.44, NIH).

### Quantitative reverse transcription-polymerase chain reaction (RT-PCR)

The mRNA levels of collagen type I, collagen type III, atrial natriuretic factor (ANF) and β-myosin heavy chain (β-MHC) were quantified using RT-PCR. The total RNA was extracted from the heart using an RNeasy Fibrous Tissue Kit (Qiagen). Complementary DNA was synthesized using a High-Capacity cDNA Reverse Transcription Kit (Applied Biomedical Inc.). The relative gene copy numbers were quantified with real-time PCR using TaqMan Gene Expression Assays (18S: 4352930; collagen type I: Mm00801606; collagen type III: Mm01254476; ANF: Mm01255748; and β-MHC: Mm00600555; Applied Biosystems, Foster City, CA). The absolute number of gene copies was normalized to the 18S rRNA gene and standardized using a standard curve.

### Statistical analysis

All of the values are expressed as the mean ± standard error. The data were compared using an ANOVA followed by Dunnett’s test for pair-wise comparisons against the “control” samples and an ANOVA followed by Tukey’s test for multiple comparisons. Correlations between two factors were analyzed by Pearson correlation. All of the statistical analyses were performed using JMP v.8 (SAS Institute, Inc., NC). *P-*values <0.05 were considered statistically significant.

## Results

### Decrease in Blood Pressure by Ang II vaccine

**Figure 1:**
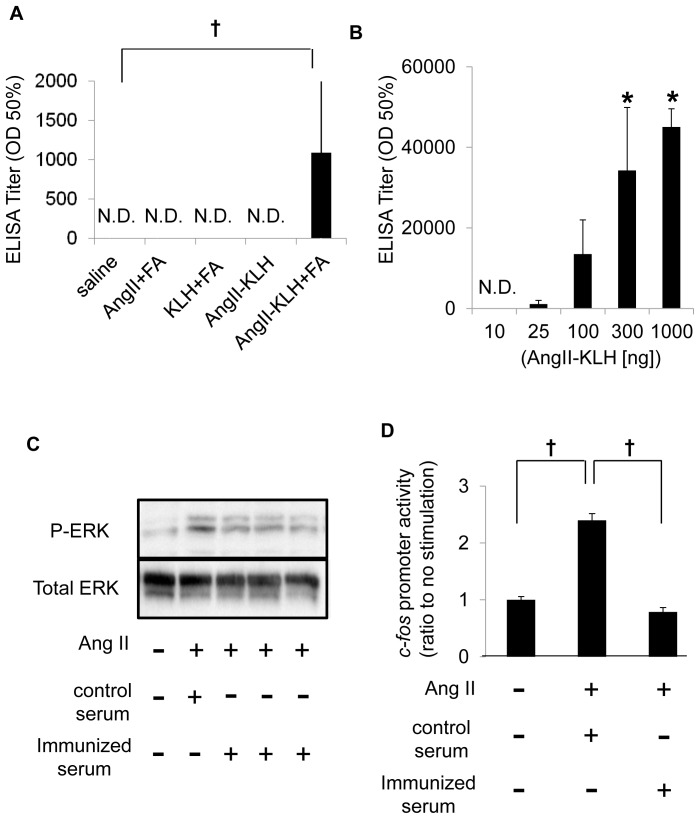
Neutralizing antibody production induced by Ang II vaccine. (A) The antibody titers in the sera of mice immunized with saline, Ang II, KLH or Ang II-KLH (dose: 25 ng/mouse) with or without Freund’s adjuvant (FA) are shown. †*P*<0.001 vs. saline. ELISA was performed using the sera of mice on day 42. (B) The serum antibody titers elicited by various doses (10, 25, 100, 300, 1000 ng/mouse) of Ang II-KLH with Freund’s adjuvant (FA). *P<0.05 vs. 10 ng. The titer is expressed as the dilution of serum giving half-maximal binding (optical density: OD 50%) ± SE of the mean. (C) Western blot using anti-phosphorylated ERK (P-ERK) and anti-ERK antibodies. The total protein was extracted from HASMCs treated with 10^−7^ M Ang II that was previously incubated with 1 % serum. (D) Promoter activity of *c-fos* was measured using luciferase activity. HASMCs were transfected with *c-fos* luciferase reporter gene and stimulated by pre-incubated Ang II (10^−7^ M) with 1 % serum of the control or immunized mice for 24 hours. The results from 3 samples are expressed as the mean of the ratio to no stimulation ± SE of the mean. ‘‘Control serum’’ indicates serum from mice immunized by KLH (1 mg, with adjuvant), and ‘‘Immunized serum’’ indicates serum from mice immunized by Ang II-KLH (1,000 ng, with adjuvant). All serum was obtained on day 42. †*P*<0.01.

We examined the antibody production of B cells by evaluating anti-Ang II antibody titers in response to vaccination with Ang II, KLH, and Ang II-KLH with or without adjuvant. Only Ang II-KLH with adjuvant induced the production of anti-Ang II antibodies at day 42 after vaccination (Figure 1A). The antibody titers in the Ang II-KLH with adjuvant group increased as the dose of the antigen increased (Figure 1B). To elucidate the efficiency of the vaccine for Ang II, we evaluated the neutralizing function of the vaccination-induced anti-Ang II antibodies in HASMCs. The sera from the mice that were immunized with Ang II-KLH (1,000 ng/mouse) showed a significant decrease in Ang II-induced ERK phosphorylation in HASMCs compared to the sera from control mice (Figure 1C). Similarly, the *c-fos* promoter activity induced by Ang II in HASMCs was decreased after incubation with the sera from immunized mice (Figure 1D). These results indicate that the induced antibody blocked Ang II signaling. In fact, the antibody has the ability not only to bind Ang II but also to neutralize the biological functions of Ang II.

**Figure 2:**
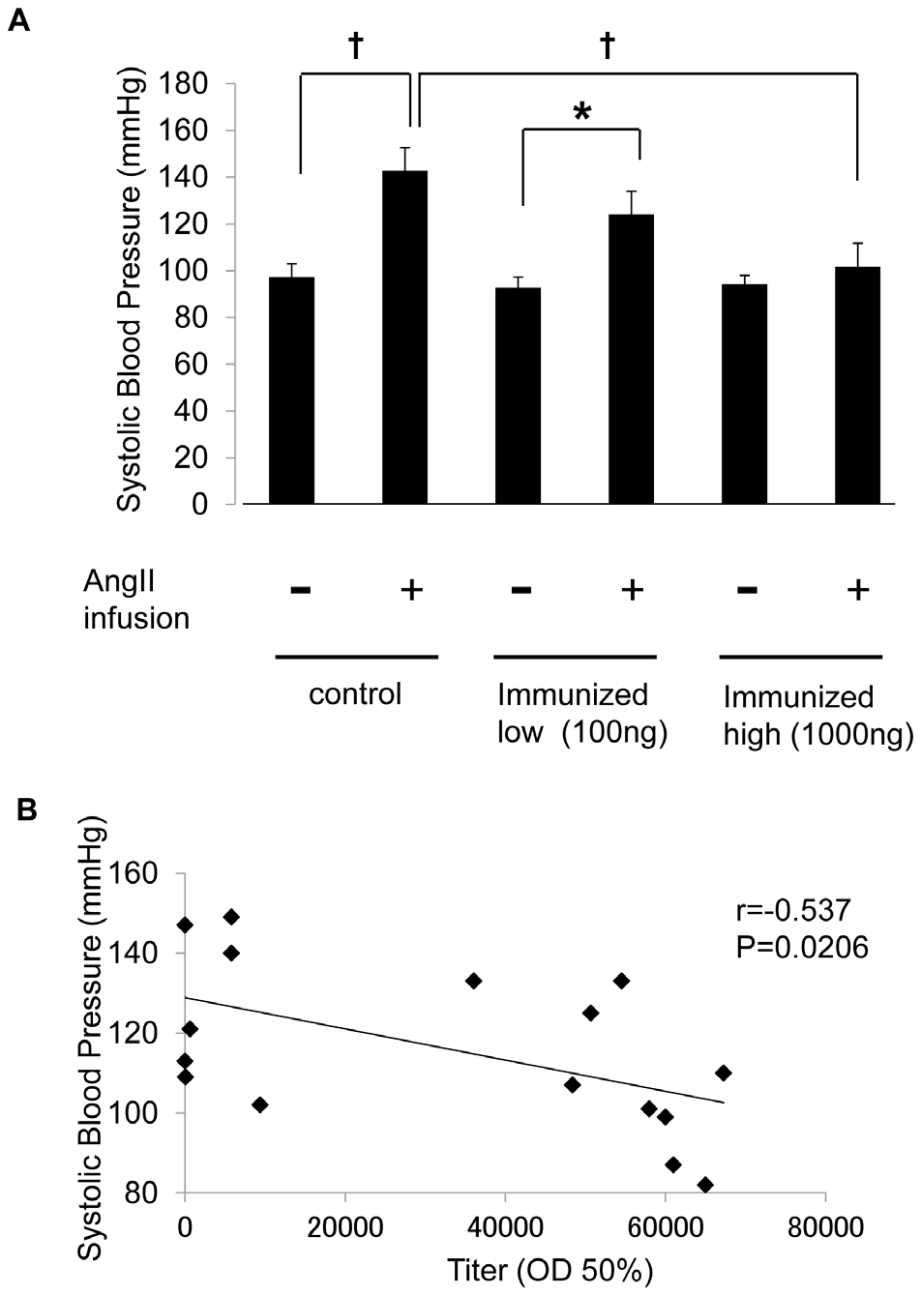
Effect of Ang II vaccine on Ang II-induced hypertension. (A) Systolic BP at steady state and under Ang II infusion on day 49. The control mice were immunized with 1 mg KLH. The experimental mice were immunized with 100ng or 1,000 ng Ang II-KLH. All groups contained 6 to 8 mice. The data are expressed as the mean systolic BP ± standard error (SE) of the mean. †*P*<0.01 *P<0.05. (B) The correlation between systolic BP under Ang II infusion and the anti-Ang II antibody titers in the sera of immunized mice. The titer is expressed as the dilution of serum giving half-maximal binding (optical density: OD 50%).

To further evaluate the effect of the immunization in mice, we examined the effect of high-dose (1,000 ng) or low-dose Ang II-KLH vaccination (100 ng) in mice using Ang II infusion. At steady state, the mice immunized with either high-dose or low-dose Ang II-KLH failed to lower BP (Figure 2A). However, systolic blood pressure was not increased in the high dose-immunized mice, while systolic BP was increased in the control mice after systemic Ang II infusion (1,000 ng/kg/min) (Figure 2A). We further analyzed the correlation between anti-Ang II antibody titer and systolic BP. A negative correlation was observed between the anti-Ang II antibody titer and systolic BP (r = 0.537, *P* = 0.0206) in the high dose- and low dose-immunized mice after Ang II infusion (Figure 2B). These results indicate that the antibody elicited by the vaccine efficiently reduced blood pressure in the Ang II infusion model.

**Figure 3:**
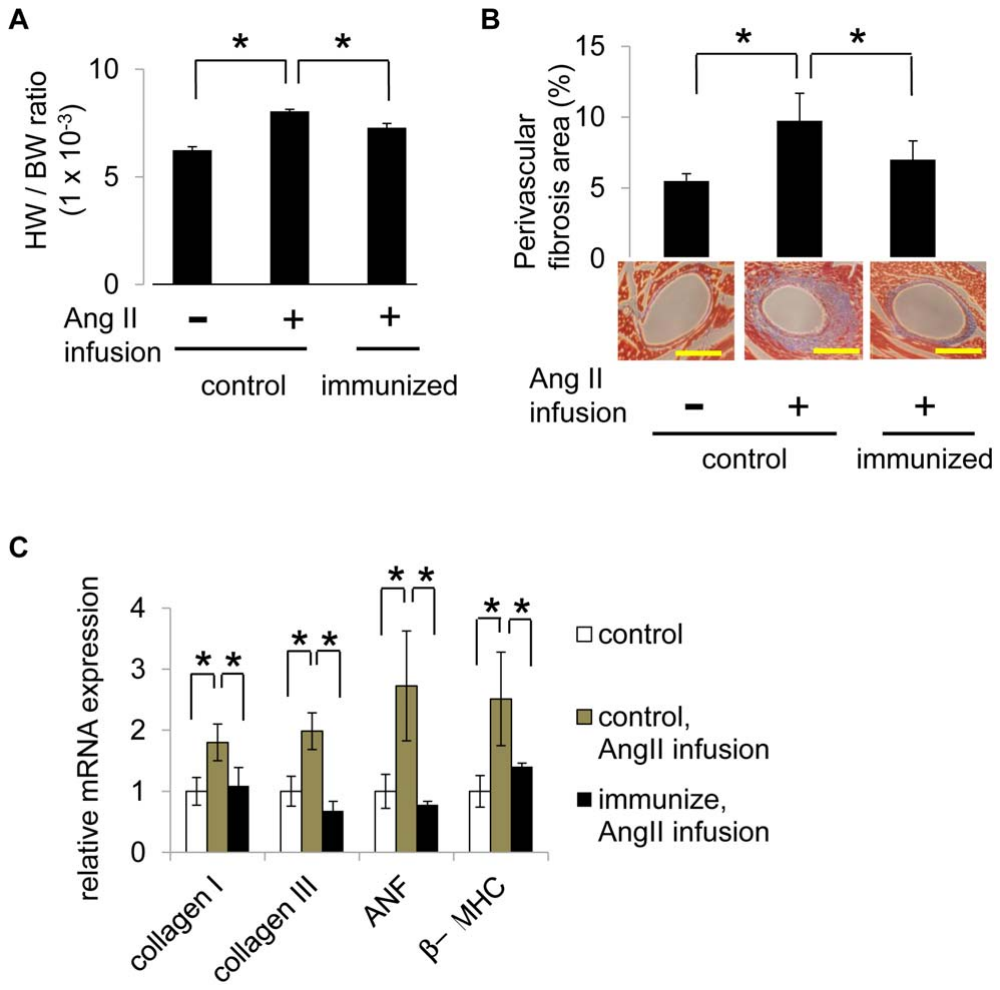
Effect of Ang II vaccine on cardiac remodeling induced by Ang II. (A) The mouse heart weights are shown. The results are expressed as the mean heart weight per body weight ± the standard error of the mean. (B) The cardiac tissue was stained with Masson trichrome and analyzed for cardiac fibrosis. Representative photographs are shown. The scale bar represents 20 µm. The results are expressed as the mean of the fibrotic area ± SE of the mean. (C) The mRNA levels of collagen type I (collagen I), collagen type III (collagen III), atrial natriuretic factor (ANF) and β-myosin heavy chain (β-MHC) were evaluated using RT-PCR. The results are expressed as the ratio of gene expression in the experimental mice to gene expression in the control mice ± SE of the mean. Control mice were immunized with KLH (1 mg, with adjuvant) and experimental mice were immunized with Ang II-KLH (1,000 ng, with adjuvant). All groups contained 6 to 8 mice on day 56. *P<0.05.

We next examined the degree of cardiac hypertrophy and fibrosis after Ang II infusion in high dose-immunized mice and control mice. Systemic Ang II treatment caused myocardial hypertrophy in control mice, whereas the immunized mice showed a significant mitigation of the Ang II-induced increase in the heart weight to body weight ratio (Figure 3A). Similarly, Ang II treatment induced perivascular fibrosis in the hearts of the control mice, while the immunized mice showed less fibrotic changes (Figure 3B). We used RT-PCR to quantify the mRNA levels of markers of fibrosis (collagen type I, III), cardiac fetal genes (ANF) and cardiac muscle hypertrophy (β-MHC). Ang II treatment significantly induced the expression levels of collagen type I and III, ANF and β-MHC, but immunized mice showed lower expression levels of these markers (Figure 3C). These results reveal that the anti-Ang II antibody induced by Ang II-KLH vaccination efficiently attenuated Ang II-induced signaling *in vitro* and Ang II-induced hypertension and cardiac remodeling *in vivo.*

### Evaluation of antibody production induced by immunization

**Figure 4:**
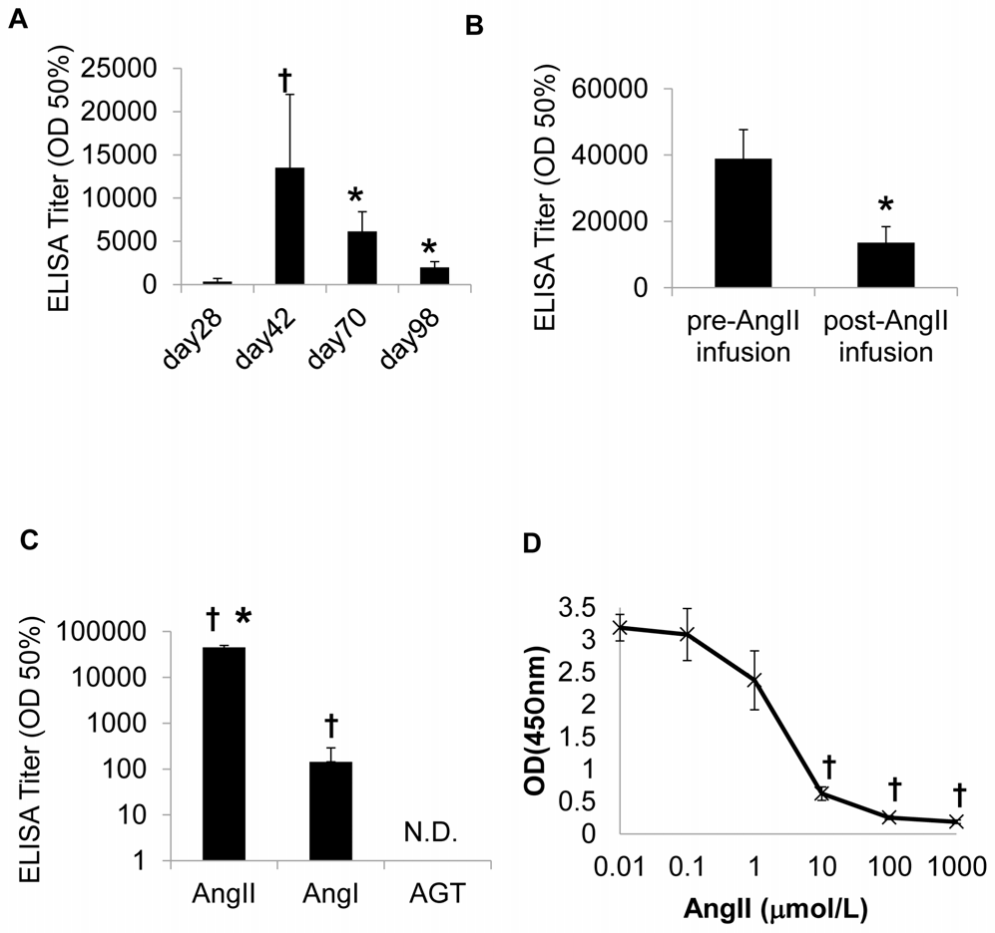
Antibody production induced by Ang II vaccine. (A) Anti-Ang II antibody titers in the sera of immunized mice on days 28, 42, 70, and 98 were examined. The results are from 6 immunized mice (100 ng Ang II-KLH with adjuvant). †*P*<0.01 vs. day 28, *P<0.01 vs. day 42. (B) Anti-Ang II antibody titer of immunized mice pre-Ang II infusion (day 42) and post-Ang II infusion (day 56). The results are from 8 immunized mice (1,000 ng Ang II-KLH with adjuvant). The titer is expressed as the dilution of serum giving half-maximal binding (optical density: OD 50%) ± SE of the mean. *P<0.01 vs. day 42. (C) Anti-Ang II, anti-Ang I, and anti-angiotensinogen (AGT) antibody titers in the sera of immunized mice (1,000??ng Ang II-KLH with adjuvant). The titer is expressed as the dilution of serum exhibiting half-maximal binding (optical density: OD 50%) ± SE of the mean. All groups contained 6 mice. “N.D.” indicates “not detected” †*P*<0.001 vs. AGT, *P<0.001 vs. Ang I. (D) The binding ability of the antibodies to native Ang II was examined by competitive ELISA. The 1000-fold diluted serum was pre-incubated with various doses of recombinant Ang II and evaluated for binding to plates coated with Ang II-BSA by means of the optical density (OD) at 450 nm. †*P*<0.001 vs. 0.01 µmol/L.

To evaluate the reversibility of the effect against Ang II by immunization, we examined the time course of anti-Ang II antibody titer at 42, 70 and 98 days after vaccination with Ang II-KLH. The antibody titer peaked on day 42 and decreased on days 70 and 98 (Figure 4A), similar to a previous report.[6], [14] To examine whether B cells could be activated by Ang II itself, we compared the antibody titer in mice prior to Ang II infusion (day 42) with the titer obtained after Ang II infusion (day 56). Importantly, the post-infusion titer was lower than the pre-infusion titer (Figure 4B). These results suggest that endogenous Ang II does not stimulate antibody production.

Regarding with the specificity of the induced antibody, anti-Ang II antibody recognized Ang I to a lesser extent but did not recognize angiotensinogen (Figure 4C). We further investigated whether the antibody could bind to native Ang II using competition ELISA because antibodies induced by small peptide-carrier conjugate may sometimes only bind to the peptide-carrier conjugate and not to the native protein. The serum that was pre-incubated with Ang II peptide showed less binding to Ang II-BSA-coated plates in a dose-dependent manner (Figure 4D), which indicates that the antibody bound not only to Ang II-BSA conjugate but also to native Ang II. These results suggest that vaccination with Ang II-KLH with adjuvant produced an antibody specific for Ang II and, to a lesser extent, Ang I in C57Bl/6 mice.

### Evaluation of T cell activation in response to immunization

**Figure 5:**
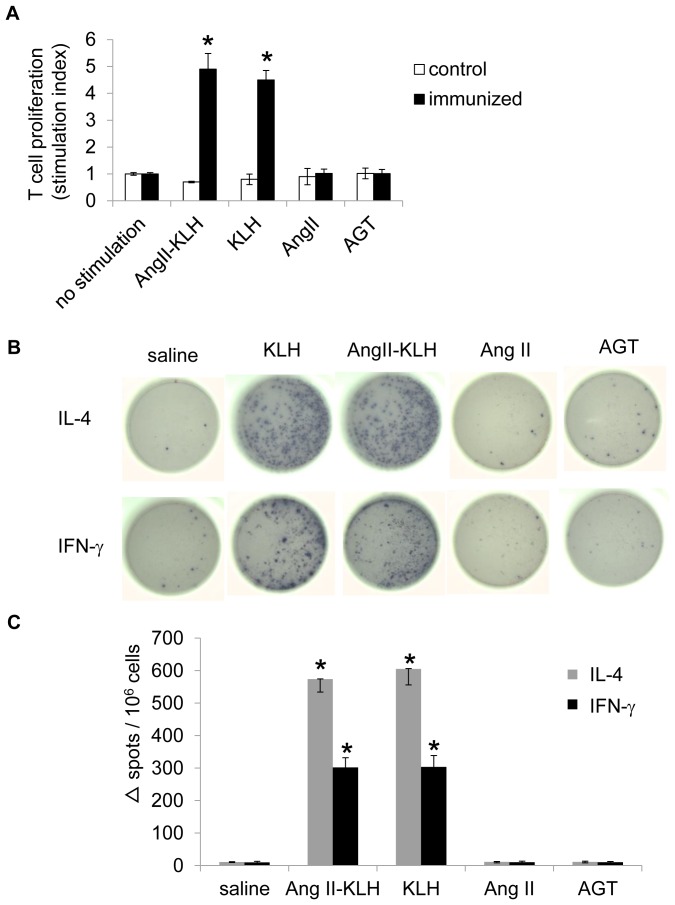
T cell activation by Ang II-KLH, KLH or Ang II. (A) T cell proliferation was determined by analyzing [^3^H]-thymidine incorporation. Splenocytes from mice on day 42 were stimulated for 36 hours with Ang II-KLH, KLH, Ang II or angiotensinogen (AGT) at a concentration of 10 µg/ml. The stimulation index is expressed as the ratio of stimulation to no stimulation. The data are expressed as the mean stimulation index ± the standard error of the mean per 10^6^ splenocytes. *P<0.001 vs. no stimulation. (B) A representative photograph from the ELISPOT assay. The ELISPOT assay detected splenocytes that produced IL-4 and/or IFN-γ? Splenocytes from mice on day 42 were stimulated for 48 hours with 10 µg/ml Ang II-KLH, KLH, Ang II or angiotensinogen (AGT). (C) The quantification of spots in the ELISPOT assay. The data are expressed as the mean number of spots ± SEM per 10^6^ splenocytes. *P<0.001 vs. saline. (a,c) The results are from 6 control mice (saline) and 6 experimental mice (1,000 ng Ang II-KLH with adjuvant).

To examine the immunization step of vaccination with Ang II-KLH with adjuvant, we finally evaluated T-cell activation using a T cell proliferation assay and ELISPOT assay on day 42 (Figure 5A). In the T cell proliferation assay, stimulation with Ang II-KLH or KLH strongly induced the proliferation of splenocytes from the mice immunized with Ang II-KLH, but it did not induce the proliferation of splenocytes from control mice administered with saline (Figure 5A). In the ELISPOT assay, stimulation with either Ang II-KLH or KLH, but neither Ang II nor angiotensinogen induced the production of IFN-γ and IL-4, with IL-4 predominating expressed (Figure 5B, 5C).

**Figure 6:**
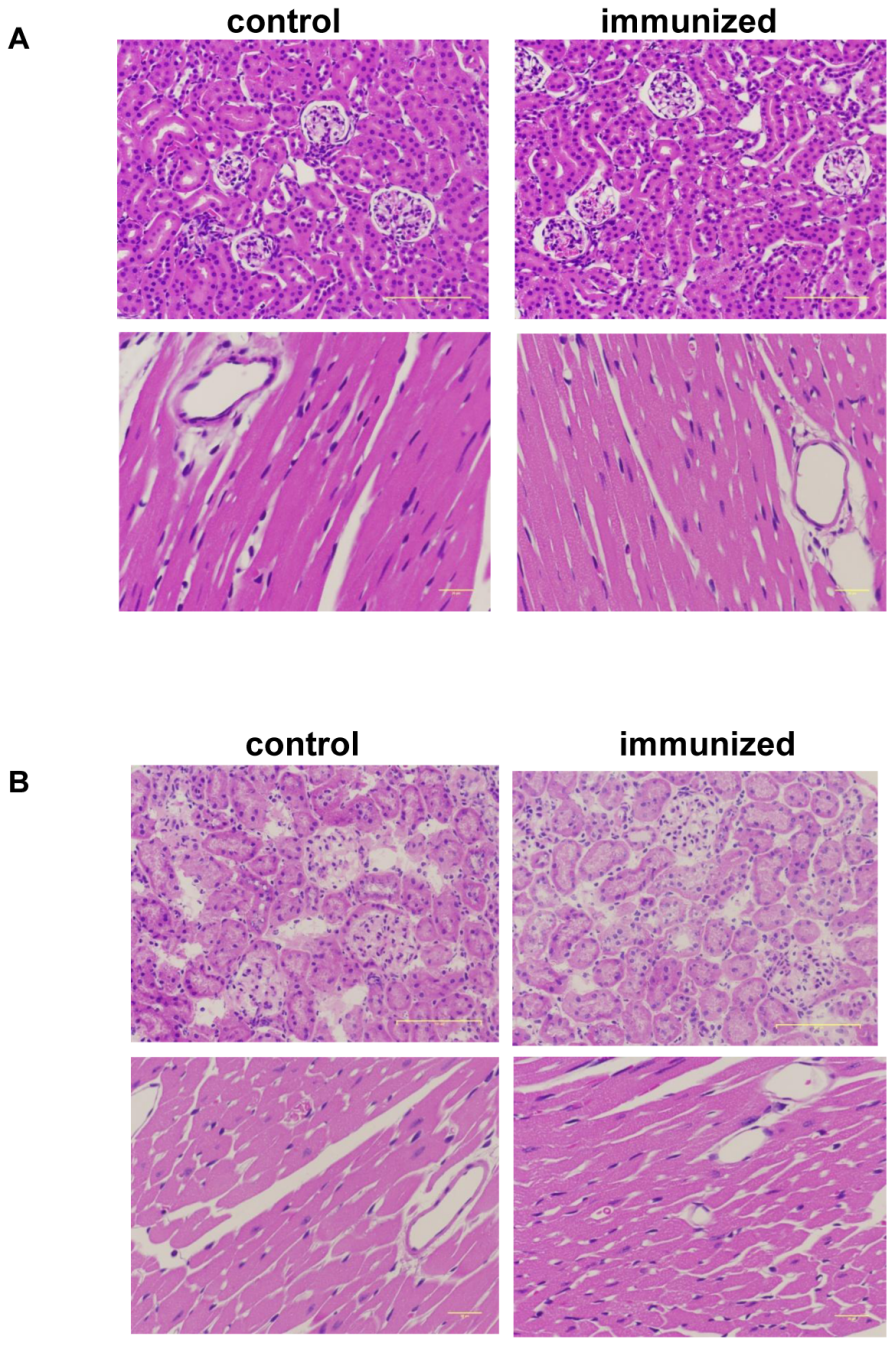
Histochemical analysis of kidney and heart after vaccination. (A and B) Kidney (upper panel) or heart with coronary artery (lower panel) was stained with H&E in control (saline) or immunized mice (1,000 ng Ang II-KLH with adjuvant) . The scale bar represents 100 µm in the upper panel or 20 µm in the lower panel. The results were examined (A) at day 42 after vaccination and (B) after Ang II infusion (day 56 after vaccination).

These data indicate that both Ang II-KLH and KLH contain adequate T-cell epitopes to induce T-cell activation but Ang II does not. These results suggest that T cells were activated by KLH, but not by Ang II or angiotensinogen. Moreover, in the ELISPOT assay, the production of IL-4 was dominant, suggesting that T-cell activation by Ang II-KLH or KLH may be skewed in the Th2 direction. The safety aspects of Ang-KLH vaccine were also confirmed by the histochemical analysis using H&E staining in the kidney and heart with coronary artery. In our case, vaccine may have some possibilities to induce the production of immune complex by the antigen-antibody reaction. For example, in the case of renin vaccine, the autoimmune nephritis was induced by the observation of immune complex depositions which can be characterized by glomerulosclerosis, mesangial hypercellularity, leucocyte accumulation, endocapilarry proliferation or immune complex deposition.[31], [32] As shown in Figure 6A, no pathological changes were observed in control or immunized group, no T-cell or macrophage infiltrations were observed in kidney or heart with coronary artery. We also performed the histochemical analysis after Ang II infusion and observed that Ang II infusion did not cause any pathological changes (Figure 6B).

### Effect of Ang II vaccine on SHR

**Figure 7:**
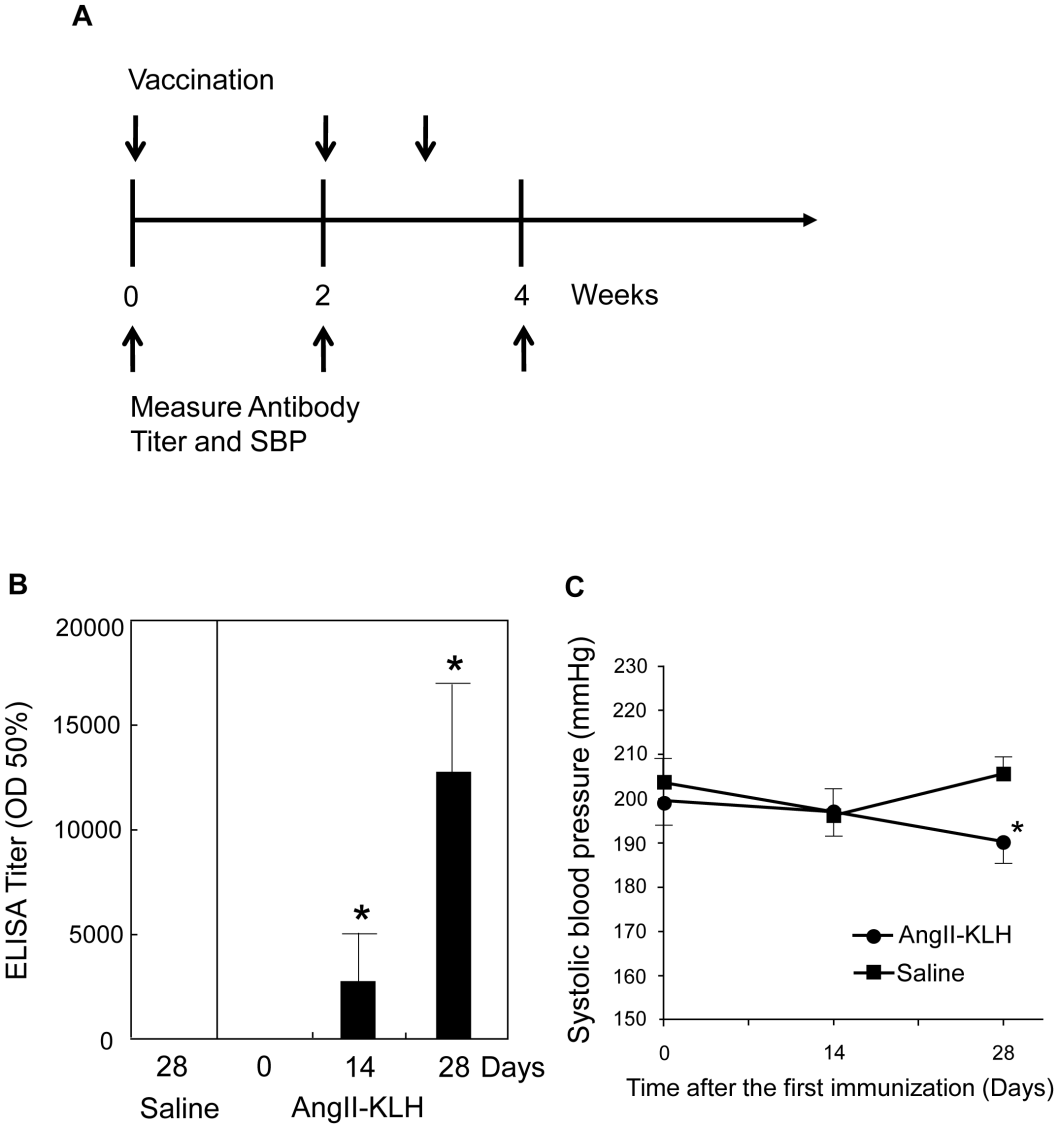
Effect of Ang II vaccine on SHR. A) Experimental protocol is shown. Ang II-KLH (Ang II-KLH group, n = 5) or saline (saline group, n = 5) were injected on days 0, 14, and 21 in twenty-four-week old male SHR. The anti-Ang II antibody titer and systolic BP were measured on days 0, 14, and 28. The Ang II-KLH group rats were immunized with 5 µg Ang II-KLH with CFA on day 0 and with 5 µg Ang II-KLH with IFA on day 14 and 21. B) Anti-Ang II antibody titers in the sera of immunized rats on days 0, 14, 28, and that of saline injected rats on day 28 were examined. *P<0.01 vs. day 0. C) Systolic BP on days 0, 14, and 28 were shown. The data are expressed as the mean systolic BP ± standard error (SE) of the mean. *P<0.05. vs. Saline.

Toward clinical application, we further evaluated the effect of AngII-KLH vaccine on relatively old SHRs (twenty-four-week old) as a therapeutic model. As shown in Figure 7A, AngII-KLH (5 µg/rat) was injected three times (on day 0, 14 and 21). The titer of anti-AngII antibody was significantly increased on day14 and further increased on day 28 after immunization (Figure 7B). As a result, systolic blood pressure was significantly decreased on day 28 after immunization (Figure 7C). These results showed us the potential effect of Ang II vaccine as a therapeutic vaccine for hypertensive patients.

## Discussion

**Figure 8:**
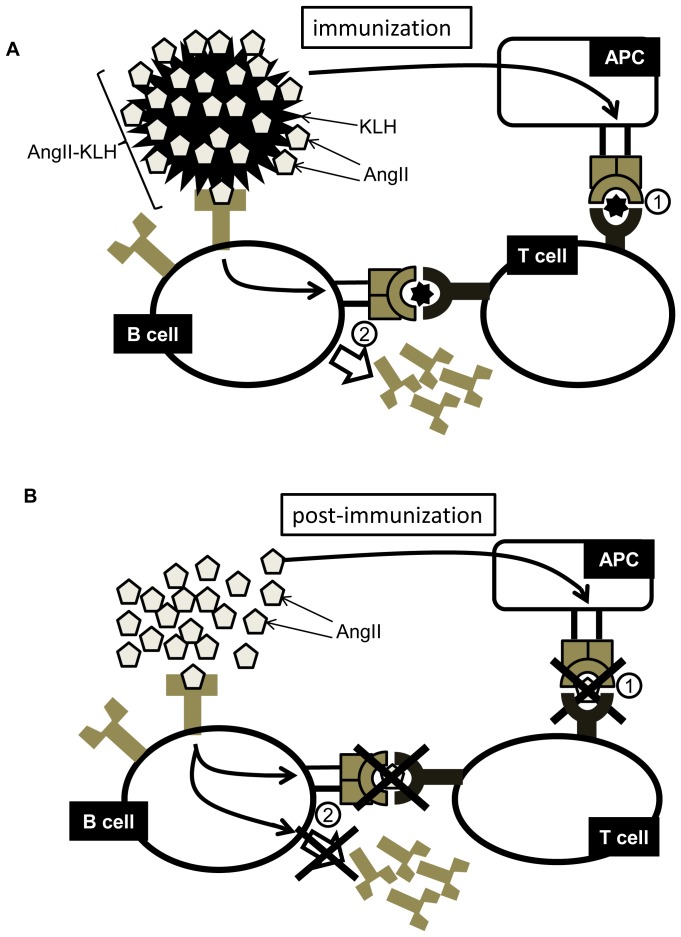
Conceptual schematic of the experiment. (A)Immunization step (Ang II-KLH: an antigen) (Step1) The antigen presenting cells (APCs) phagocytose the Ang II-KLH conjugate and present a T cell epitope of KLH to T cells through the major histocompatibility complex (MHC). T cells recognize it through T cell receptors and become activated. (Step2) B cells specific to Ang II (pentagons) differentiate to plasmacytes and proliferate with the help of activated T cells. Then, B cells produce anti-Ang II antibody. (B)Post-Immunization step (response to Ang II) (Step1) The APCs do not present the T cell epitope of Ang II to T cells. Therefore, T cells do not recognize it and are not activated by Ang II. (Step2) B cells specific to Ang II (pentagons) differentiate and proliferate in response to Ang II. Therefore, Ang II does not stimulate the production of anti-AngII antibody.

Here, an Ang II peptide vaccine effectively reduced BP and prevented cardiovascular complications such as cardiac fibrosis by stimulating anti-Ang II antibody production without severe adverse effects. Our working hypothesis about Ang II vaccine therapy was evaluated in each step of the study (see Figure 8). As shown in Figure 8A, mice are immunized with Ang II-KLH and adjuvants to circumvent T cell tolerance. As an immunization phase, antigen-presenting cells (APCs) phagocytose Ang II-KLH and present a T cell epitope of Ang II-KLH to T cells through the major histocompatibility complex (MHC), and T cells recognize it through the T cell epitope and become activated (i.e., differentiate to effector T cells) (step 1). B cells, specific to Ang II phagocytose Ang II-KLH and present the T cell epitope of Ang II-KLH to T cells through MHC. Then, B cells differentiate to plasmacytes and produce antibodies with the help of activated T cells (effector T cells) (step 2).

In this study, we confirmed that the vaccine with Ang II-KLH and adjuvants efficiently induced an antibody titer of Ang II and induced T cell activation. Generally, antigens must contain both B-cell and T-cell epitopes. We and others have confirmed that antibodies against Ang II have been produced successfully[14], confirming the existence of a B-cell epitope in Ang II. As for T-cell epitopes, we perform a T cell proliferation assay and ELISPOT assay. The results show that Ang II-KLH and KLH induced T cell activation, but Ang II did not (Fig. 4–5), which may suggest that only KLH contains a T-cell epitope. Therefore, eight amino acids of Ang II could activate B cells but not T cells in our animal model. This situation is reflected in the relationship between hapten and its carrier, in which hapten has only a B-cell epitope and the carrier possesses a T-cell epitope.[33] Based on this finding, we used Ang II and KLH as hapten and its carrier to successfully produce an antibody against Ang II which was assisted by helper T-cell activation.

The immune response against pathogens comprises factors that activate both T cells and B cells. It lasts until the invaders are removed. However, the “natural boost” for most autoimmune diseases is generated by a self-antigen, and leads to the maintenance of chronically high antibody titers because the factors activating T cells and B cells are inherent to the body and cannot be cleared.[34] Therefore, we also investigated the response to endogenous Ang II after immunization, as shown in Figure 8B. In this case, APCs do not present T cell epitope of Ang II to T cells. Hence, endogenous Ang II does not activate T cells (i.e., cause them to differentiate to effector T cells) (step 1). Because B cells do not present the T cell epitope of Ang II to T cells, B cells are not stimulated because of the lack of the help of the effector T cells. Although B cells are sometimes stimulated by an antigen without the help of T cells, Ang II does not directly stimulate B cells (step 2). Similar to a previous report[14]_ENREF_14_ENREF_14_ENREF_14, our study showed that the Ang II vaccine did not maintain a high titer after the last boost (Fig. 4A). Furthermore, a large amount of Ang II administration did not induce antibody production in immunized mice (Fig. 4B). This result implies that endogenous Ang II does not induce the activation of T cells or the production of anti-Ang II antibodies, improving its safety for clinical use.

The use of vaccines targeting self-antigens has recently been reported for cancer [2], rheumatoid arthritis [1], Alzheimer’s disease [3]–[12], [25], [26], hypertension [6], [13], [14], and dyslipidemia.[35] Because safe and effective drug therapies have already been established for several of these conditions, adverse vaccine effects should be carefully considered, especially in hypertension and dyslipidemia. The key goals of this approach are avoiding T-cell activation in response to self and the reversible inhibition of the target molecule. The Alzheimer’s disease clinical trial was halted because the participants developed aseptic meningoencephalitis due to an autoimmune response.[4], [8]–[10] Because amyloid beta-induced T-cell activation was considered responsible for the adverse event, we examined whether Ang II had the ability to trigger a T-cell-mediated immune response using T cell proliferation and ELISPOT assays. Splenocytes from the immunized mice responded to Ang II-KLH and KLH, but not to Ang II (Fig. 5A–C), which showed that Ang II does not induce T cell activation. In another case, vaccine may have some possibilities to induce the production of immune complex by the antigen-antibody reaction. For example, in the case of renin vaccine, the autoimmune nephritis was induced by the observation of immune complex depositions same as Lupus nephritis or IgA nephropathy. [31], [32] In this study, immunized mice did not show any findings even after Ang II infusion, which suggest that Ang II vaccine did not induce autoimmune nephritis unlike renin vaccine in mice. Therefore, we suggest that this vaccine does not induce an autoimmune disease because KLH is a completely “non-self” antigen in the human body and Ang II-KLH only exists during immunization.

In conclusion, we propose that this Ang II vaccine is safe, does not carry the risk of an anti-Ang II T cell-mediated autoimmune response, and effectively induces the production of anti-Ang II antibody. In this study we employed Ang II infusion in mice after the antibody titer peaked, which corresponds to preventive therapy. As a therapeutic model, the Ang II vaccine was also effective at reducing blood pressure in SHR[14]. In this study, we successfully showed that Ang II vaccine was also effective at reducing blood pressure in relatively old SHR. Although further studies are necessary to develop the Ang II vaccine into a real treatment for hypertensive patients, future modifications supporting the clinical application of this vaccine may identify a novel immunotherapy for hypertension.
